# A Molecular Fluorescent Probe for Targeted Visualization of Temperature at the Endoplasmic Reticulum

**DOI:** 10.1038/srep06701

**Published:** 2014-10-21

**Authors:** Satoshi Arai, Sung-Chan Lee, Duanting Zhai, Madoka Suzuki, Young Tae Chang

**Affiliations:** 1 Department of Chemistry, National University of Singapore, MedChem Program of Life Sciences Institute, National University of Singapore, 3 Science Drive 3, Singapore 117543, Republic of Singapore; 2 Consolidated Research Institute for Advanced Science and Medical Care, Waseda University, 513 Wasedatsurumaki-cho, Tokyo 162-0041, Japan; 3 Laboratory of Bioimaging Probe Development, Singapore Bioimaging Consortium, Agency for Science, Technology and Research (A*STAR), Biopolis, Singapore 138667, Republic of Singapore; 4 WASEDA Bioscience Research Institute in Singapore (WABIOS), 11 Biopolis Way, #05-02, Helios, Singapore 138667, Republic of Singapore; 5 Organization for University Research Initiatives, Waseda University, #304, Block 120-4, 513 Wasedatsurumaki-cho, Shinjuku-ku, Tokyo, 162-0041, Japan

## Abstract

The dynamics of cellular heat production and propagation remains elusive at a subcellular level. Here we report the first small molecule fluorescent thermometer selectively targeting the endoplasmic reticulum (ER thermo yellow), with the highest sensitivity reported so far (3.9%/°C). Unlike nanoparticle thermometers, ER thermo yellow stains the target organelle evenly without the commonly encountered problem of aggregation, and successfully demonstrates the ability to monitor intracellular temperature gradients generated by external heat sources in various cell types. We further confirm the ability of ER thermo yellow to monitor heat production by intracellular Ca^2+^ changes in HeLa cells. Our thermometer anchored at nearly-zero distance from the ER, i.e. the heat source, allowed the detection of the heat as it readily dissipated, and revealed the dynamics of heat production in real time at a subcellular level.

Sustaining body temperature is of fundamental importance in endothermic animals^1^,^2^. At a subcellular level, the mitochondrion is a well-known organelle for non-shivering thermogenesis^3^. More recently, the sarco/endoplasmic reticulum has also garnered attention as one of the organelles responsible for heat production, mediated by the sarco/endoplasmic reticulum Ca^2+^-ATPase (Serca) pump^4^. Temperature measurements within the compartmentalized interior structure of interest, i.e. target-organelle thermometry, are important to monitor the precise dynamics of heat production in cells. For intracellular thermometry, an optical probe called a fluorescent thermometer, which reports small temperature differences as a fluorescent signal, presents a promising tool in terms of spatial resolution^5^. However, it is still challenging to smoothly control the intracellular locations of these thermometers including dye-doped polymeric particles^6^,^7^, quantum-dots^8^, upconverting particles^9^, nanodiamond^10^ and nano-gel particles^11^, as they either require a micro-injection or often have aggregation problem along with uneven endocytosis and escape from endosomes. Fluorescent proteins as thermosensors^12^,^13^ prevail when it comes to targeting ability to organelles, however, their low sensitivity to temperature is considered problematic. Furthermore, laborious procedures such as the construction of a virus vector are unavoidable for effective expression of a fluorescent protein. Here, we report a small organic molecule fluorescent thermometer with targeting ability to ER and high thermosensitivity. A small fluorescent molecule demonstrating cell permeability and robust stability is a convenient tool for bio-imaging applications, with the added advantage over nanoparticles of even distribution in cells. In recent years, we have a generated diversity oriented fluorescent library (DOFL) through combinatorial synthesis by the modification of side chains of the different fluorescent dye backbones^14^. To date, the DOFL approach has found fluorescent probes distinguishing cell types and/or labeling cellular components^15^,^16^. By expanding this strategy, we screened the DOFL for temperature sensitive dyes and also those targeting the ER in live cells. By using an ER targeting small molecule thermometer, we demonstrate the targeted visualization of temperature at the ER in a single HeLa cell.

## Results

### Discovery of a fluorescent thermometer targeting the ER

**Figure 1:**
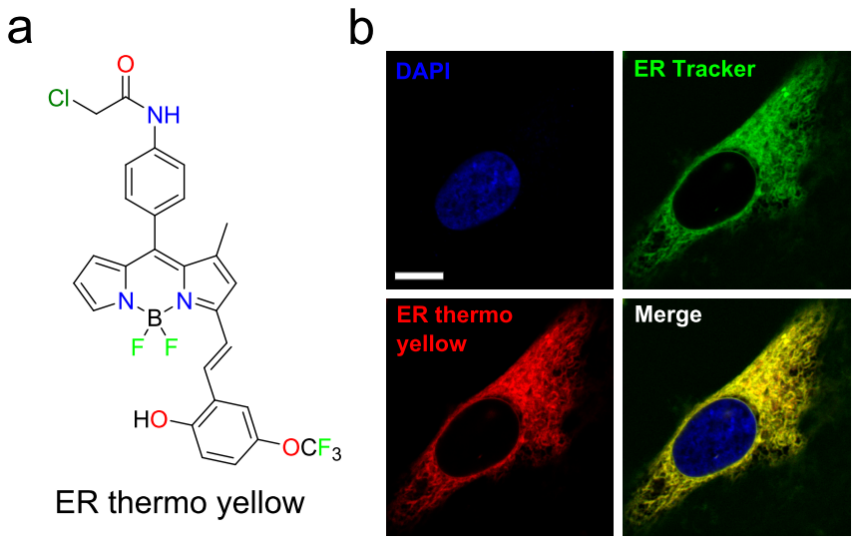
ER targetable fluorescent thermometer. (a) Chemical structure of ER thermo yellow. (b) Co-localization of ER thermo yellow and ER tracker green. HeLa cells were stained with DAPI, ER tracker green, and ER thermo yellow. The images were captured using a confocal microscope equipped with x 60 objective lens. Pearson's correlation coefficient = 0.90; Scale bar, 5 μm. The images are shown in pseudo colors.

Currently several small dyes, such as europium (III) thenolyltrifluoro-acetonate (Eu-tta) and 7-nitrobenz-2-oxa-1,3-diazol-4-yl (NBD), have been reported as optical thermometers that function in live cells^17^,^18^. The temperature sensitivity of these dyes is around 2%/°C under physiological conditions and stains cellular membranes non-specifically. We examined the temperature sensitivity of a total of 1,500 molecules generated through combinatorial synthesis by the modification of side chains of different fluorescent dye backbones^19^. By using a temperature controlling device equipped with a microplate reader, temperature sensitivity was evaluated by repeated heating (37°C) and cooling (32°C) of 96 well plates loaded with these dyes in HEPES solutions, which were kept inside the plate reader. By heating the plate, the fluorescence intensity of most dyes decreased, following the principle that the non-radiative process without emitting fluorescence is preferred at higher temperatures. Under cooling conditions, the intensity returned to the original level before heating, contingent on the dye not precipitating or degrading. The temperature sensitivity of each dye was calculated from the change of fluorescence intensity (Supplementary Scheme S1). We selected 12 dyes with a temperature sensitivity above 1%/°C and further evaluated this sensitivity in live HeLa cells. Taking into consideration photostability, brightness, and temperature sensitivity inside cells, we finally selected a compound (BDNCA346) that localizes specifically to the ER (Supplementary Scheme S2; Fig.S1-S6) and displayed the best temperature sensitivity. We refer to this compound as ER thermo yellow (λ_abs_/λ_em_ = 559/581, Fig. 1).

### Evaluation of the temperature sensitivity of dyes in live HeLa cells

**Figure 2:**
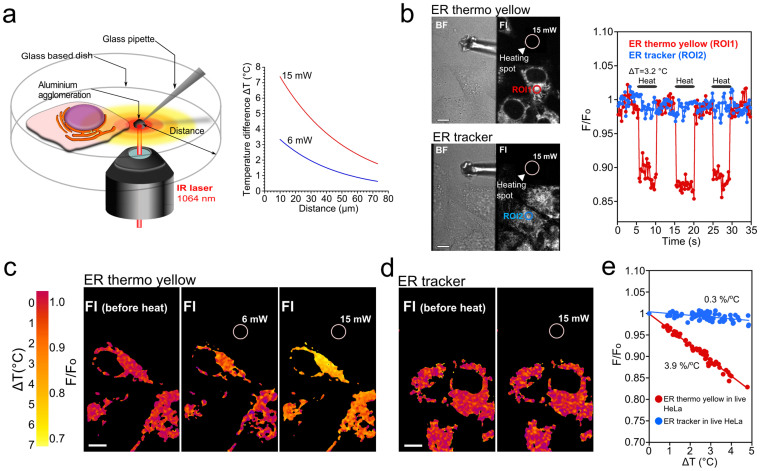
Evaluation of the temperature sensitivity in live HeLa cells. (a) Schematic representation of the evaluation system based on a confocal laser scanning microscope with an infrared laser. An inset graph shows that the concentric temperature gradient at a microscopic scale. This schematic was originally drawn by one of the authors, S. Arai. (b) (left panels) the regions of interest (ROI) ROI1 and ROI2 were the same distance from a heating spot generated by an infrared laser (15 mW). The temperature rises 3.2°C at ROI1 and ROI2 from a base temperature of 37°C. (right graph) (c–d) The fluorescence images obtained during heating were divided by the image before heating (F/F_o_). (c) The temperature mapping of ER thermo yellow was obtained by different laser powers, such as 6 and 15 mW. (d) ER tracker was less sensitive even at a higher laser power of 15 mW. (e) The temperature sensitivity of ER thermo yellow (n = 63) and ER tracker (n = 78) was estimated. Δ*T* was determined from the distance between the heating spot and the ROI as shown in Fig. 2a. The difference (ΔF/F_o_) was plotted against Δ*T*. n is the number of regions of interests. The numbers of cells range from 8–10. Scale bar, 10 μm.

The temperature sensitivity of dyes in single live cells was evaluated in a microscopy system utilizing a near infrared laser (1064 nm, Fig. 2a)^7^. The laser is focused on the agglomeration of aluminum powders attached onto the tip of a glass microneedle. This generates a local temperature gradient at a microscopic scale, while maintaining the extracellular medium at 37°C. The resulting temperature rise depends on both the distance from the focus spot and the laser power (Fig. 2a, right; Supplementary Fig. S7). When single HeLa cells stained with ER thermo yellow were exposed to the temperature gradient, the fluorescence intensity decreased (Fig.2b, ROI1). The shutter was then closed, the heat diffused out to the surrounding medium, and the fluorescence intensity returned to the original level prior to heating within two camera frames (440 msec). We obtained the ‘square wave' in response to repeated heating and re-cooling cycles. The temperature gradient was also visualized within single cells during heating (Fig. 2c). The normalized fluorescence intensity of ER thermo yellow (F/F_o_) was plotted against the temperature difference (Δ*T*, Fig. 2e). Within the range of Δ*T* from 0°C to 5°C, the calculated temperature sensitivity was 3.9%/°C. To the best of our knowledge, this sensitivity is the highest ever among current intensiometric fluorescent thermometers of a single molecule type capable of working in aqueous conditions^5^. A commercially available ER tracker (λ_abs_/λ_em_ = 587/615) hardly showed a square wave, and was therefore identified as being insensitive to the temperature change (Fig. 2b, ROI2; 2e).

### Validation of the temperature sensitivity of ER thermo yellow in fixed HeLa cells and various other live cells

**Figure 3:**
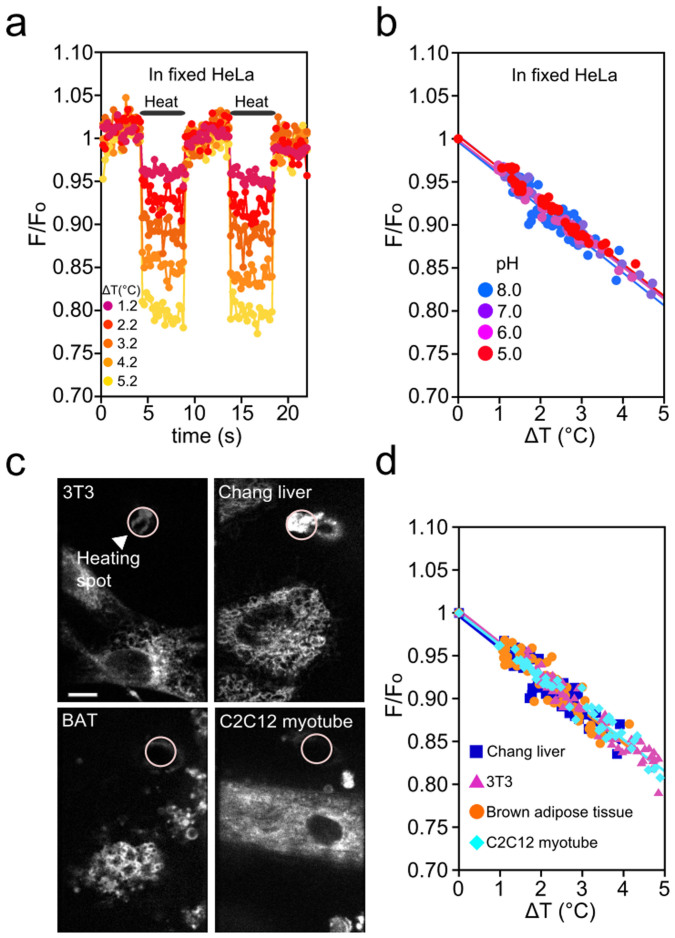
Validation of the temperature sensitivity of ER thermo yellow. (a) In fixed HeLa cells, the fluorescence of ER thermo yellow also showed the square wave pattern in response to a temperature difference. (b) The temperature sensitivities in 20 mM HEPES buffer solutions (pH = 5.0, 6.0, 7.0 and 8.0) were 4.0%/°C (R^2^ = 0.96, n = 41), 3.7%/°C (R^2^ = 0.97, n = 41), 3.7%/°C (R^2^ = 0.99, n = 38), 3.8%/°C (R^2^ = 0.98, n = 44), in order. (c) The pink circle is the tip of the needle at a heating spot. Scale bar, 10 μm. (d) The temperature sensitivity in Chang liver, NIH3T3, Brown adipose tissue (BAT), and C2C12 myotube were 3.8%/°C (R^2^ = 0.85, n = 56), 3.8%/°C (R^2^ = 0.94, n = 58), 3.8%/°C (R^2^ = 0.81, n = 50), 3.7%/°C (R^2^ = 0.96, n = 47), respectively. In Fig. 3b,d, Δ*T* was determined from the distance between the heating spot and the ROI. The ΔF/F_o_ was plotted against Δ*T*.

We further examined the temperature sensitivity of ER thermo yellow in fixed HeLa cells by using the same evaluation system employed in live cells. ER thermo yellow has a thiol reactive moiety, which allows covalent attachment to biomacromolecules at the ER. As expected, ER thermo yellow maintained fluorescence after fixation. Consequently, we obtained the same square wave pattern and temperature sensitivity as in live HeLa cells (Fig. 3a, b, 3.9%/°C at pH 7.0). Additionally, we tested whether calcium and pH affect the fluorescence intensity of ER thermo yellow. In particular, free Ca^2+^ concentration ([Ca^2+^]) is an important factor as the ER is one of the key organelles involved in the dynamic regulation of [Ca^2+^] in the cytosol. Genetically-encoded calcium indicators expressed in the ER revealed that [Ca^2+^] in the ER changes only by a few hundred micromolars even with the addition of ionomycin (1 μM), which causes calcium influx from the extracellular medium^20^,^21^. The fluorescence intensity of ER thermo yellow was unaffected by [Ca^2+^] (0–800 μM, Supplementary Fig. S9). pH changes within a physiological range (5.0–8.0)^22^ also showed no effect in the temperature sensitivity of ER thermo yellow (Fig. 3b).

The temperature sensitivity also remained constant in the different cell lines and primary cells that we examined - these include skeletal muscle cells and brown adipocytes (Fig. 3c and d) that have thermogenic functions *in vivo*. The list of cells we examined is not exhaustive and therefore we suggest that the temperature sensitivity of ER thermo yellow should be determined for each type of cell of interest.

### Visualization of Ca^2+^ activated heat production at the ER in a single HeLa cell

**Figure 4:**
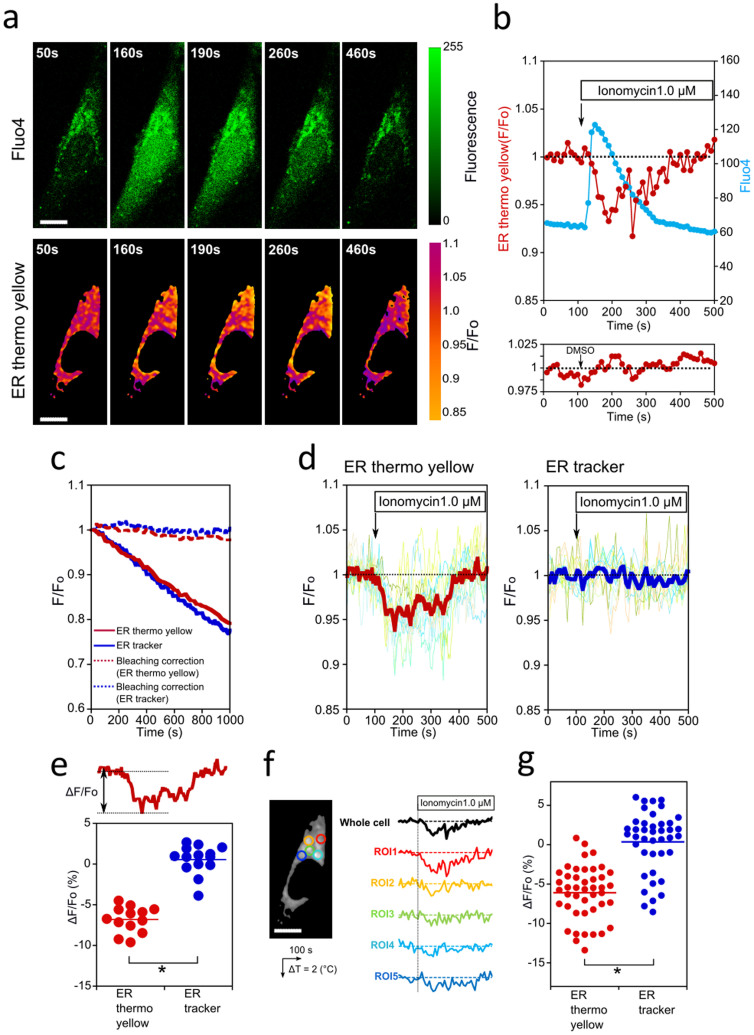
Observation of Ca^2+^ activated heat production at a single cell level. (a) Ionomycin was added to the imaging medium at a final concentration of 1.0 μM. Elapsed time is shown to the top left of each image (Scale bar, 5 μm). (b) Time courses of the fluorescence emitted by ER thermo yellow (red line) and Fluo 4 (blue line) are shown. For the control experiment, the time course of ER thermo yellow was monitored after the addition of DMSO (the medium containing 0.05%(v/v)), which is shown in the lower panel. (c) The photobleaching time course of ER thermo yellow (solid red line) and ER tracker (solid blue line). Photobleaching was corrected by fitting with a single exponential curve (dash lines). See Supplementary Figure S10 and S12. (d) The average of the normalized fluorescence intensity was calculated from a total cell area with respect to each cell. The time course of the normalized intensity was plotted against time; ER thermo yellow (left panel, N = 13) and ER tracker as a temperature insensitive dye (right panel, N = 14). The thick line represents the average of the cells. (e) To support the visual comparison in (d), the difference (ΔF/F_o_) was calculated from each cell based on the data corresponding to the data as shown in (d). Regarding ER thermo yellow, the ΔF/F_o_ value was −6.8 ± 1.5% corresponding to 1.7 ± 0.4°C (means ± SD), while ER tracker was 0.6 ± 1.7%. *P<0.001, Student's *t*-test. (f) Profiles of heat production at the different ROIs within the same cell. (g) According to the method shown in (e), the ΔF/F_o_ value varying from spot to spot was analyzed in both ER thermo yellow and ER tracker. The average of ER thermo yellow was −6.1 ± 3.1% (N = 6, n = 44) corresponding 1.6 ± 0.8°C (means ± SD), while that of ER tracker was −0.4 ± 3.8% (N = 5, n = 41). *P<0.001, Student's *t*-test. Scale bar, 5 μm. n is the number of ROIs. N is the number of cells. Photobleaching for all data was corrected by fitting with a single exponential curve as shown in (c).

Finally, we demonstrate the ability of ER thermo yellow to monitor heat production at a single cell level. The Ca^2+^ ionophore, ionomycin, elevates cytosolic [Ca^2+^], thus changing the [Ca^2+^] gradient between the cytoplasm and ER, and may cause uncoupling of the SERCA pump, so generating heat^8^,^23^,^24^. When ionomycin was added to HeLa cells (stained with both ER thermo yellow and Fluo 4) during microscopic observations, the fluorescence intensity of Fluo 4 increased, whilst that of ER thermo yellow decreased (Fig. 4a and Supplementary Movie). While the fluorescence intensity of Fluo 4 started to decrease homogeneously, that of ER thermo yellow returned to the original level prior to the addition of ionomycin (Fig. 4b). These results indicate that heat production at the ER begins with the increase of cytosolic [Ca^2+^] and that the rate of heat production gradually decreases. The ER tracker showed little change in fluorescence intensity upon the addition of ionomycin (Fig. 4d, right graph). We estimated Δ*T* from cell to cell individually as shown in Fig. 4d, e. The difference in fluorescence intensity of ER thermo yellow produced by ionomycin was −6.8 ± 1.5%, corresponding to 1.7 ± 0.4°C (mean ± standard deviation (SD)), while the ER tracker showed a change of 0.6 ± 1.7%. The ΔF/F_o_ distribution of ER thermo yellow was similar to that of the less thermosensitive ER tracker, which was used as a control dye (F = 1.23908 < F_0.025, 14, 13_ = 2.660177 examined by the *F*-test at a 0.05 significance level). This means that the Δ*T* distribution (SD = 0.4°C) of ER thermo yellow can be attributed to the intrinsic measurement error, and that the temperature measurement was accurate with a SD of 0.4°C from whole cell analysis. It is therefore apparent that heat production did not significantly vary from cell to cell. At the single cell level, heterogeneous heat production is likely to occur even within the cell shown in Fig. 4f. However, the *F*-test showed ER thermo yellow (−6.1 ± 3.1%) and ER tracker (−0.4 ± 3.8%) to have equal variance (F = 1.323882 < F_0.025, 41, 44_ = 1.672165 at a 0.05 significance level). The difference in Δ*T* at the single cell level (SD = 3.1%, or 0.8°C) is not attributed to the heterogeneous change in temperature caused by Ca^2+^ shock but the intrinsic measurement error (Fig. 4g). The accuracy of the temperature measurement was also determined to be 0.8°C in a spot by spot analysis, with each spot having a radius 1.6 µm.

## Discussion

In this study, we propose a way to evaluate the temperature sensitivity of dyes in both live and fixed cells by using a fluorescence microscope coupled with a near infrared laser. It is standard practice in the development of fluorescent thermometers that temperature sensitivity is evaluated under cellular conditions. The molecular environment of the thermometer, such as solvation and degrees of freedom, is quite different in cells from that of buffer conditions^25^. This is exemplified by the fact that the sensitivity of ER thermo yellow in HEPES buffer solution was 1.7%/°C (Supplementary Fig. S8), which differs from that in live and fixed HeLa cells (3.9%/°C). This is not surprising as the temperature dependency of a fluorescent molecule is affected by the rotational freedom of its substituent groups^26^. The binding of ER thermo yellow to some biomacromolecules at the ER is likely to alter its thermosensitivity, although its precise binding partners remain unknown. We also examined the thermosensitivity of ER thermo yellow in fixed cells, which has rarely been done for other fluorescent thermometers previously reported. We consider the comparison of temperature sensitivity between live and fixed cells provides more accurate information on the thermosensitivity of ER thermo yellow as changes in intracellular temperature in living cells alter a host of other factors that are likely to affect the fluorescence of dyes and lead to the under- or overestimation of temperature sensitivity. These factors include cytosolic Ca^2+^ concentration^27^,^28^, cellular viscosity^29^, and enzymatic activities^30^.

We validated the temperature sensitivity of ER thermo yellow in fixed HeLa cells by changing the temperature in the exterior medium. This is a straightforward way to change the temperature of a whole cell, which differs from the approach taken to create a local rise in temperature. Nonetheless, this different approach provided almost the same temperature sensitivity values (4.0%/°C) in fixed HeLa cells (Supplementary Fig. S11). This demonstrates that temperature sensitivity can be validated with a common fluorescence microscope equipped with a circulation chamber.

Recently how we deal with intracellular temperature has been a topic of heated debate^31^,^32^. We therefore stress that the current results do not indicate that the internal temperature rise of above 1°C induced by Ca^2+^ shock occurs throughout the whole cell but rather at specific organelles. The three-dimensional heat conduction equation can be described as follows: 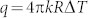
where *R* denotes the radius of the sphere, *q* the heat per second (W), *k* thermal conductivity (W/m/K) and Δ*T* temperature difference. Given that the cell has a 10 µm sphere of water (0.6 W/m/K), a *q* of around 100 µW is needed to maintain a temperature rise of a whole cell by 1°C. However, this does not agree with the data obtained from the microcalorimetry studies in the field of thermal biology^33^,^34^. These studies indicate that an individual cell releases heat (*q*) in the order of pico to nanowatts, although it should be taken into account that these experiments are performed using cell suspensions that may therefore be a mixture of dead and live cells. Our thermometer is placed inside the compartmentalized interior of a cell, at a ‘nearly zero' distance from the heat source, which enables the accurate measurement of local and small changes in heat production. Until now, Yang et al.^8^ and Takei et al.^35^ determined Δ*T* induced by Ca^2+^ shock to be around 1.8°C inside the cell, which is in accordance with our results. The nanothermometers of Yang et al. also revealed that Δ*T* was heterogeneous with a broad distribution. They discussed that this heterogeneity was due to variation in distances between thermometers and heating spots. In contrast, ER thermo yellow localizes inside the ER, i.e. where the organelle heat source resides, at a distance from the heat source that is nearly zero. As judged from data variation from spot to spot within a single cell, we did not detect heterogeneity in Δ*T* (examined by the *F*-test, Fig. 4f), supporting their discussion. More recently, the nano-gel thermosensor has emerged as a pioneering work yet the distance of the thermometer from the heating spot is hard to estimate due to the limitation of spatial resolution. Furthermore, it takes a long time (approximately 1 min.) to take one image due to the restrictions of life-time imaging microscopy, which may not be ideal for tracking the dynamics of heat production. We propose ER thermo yellow as an easy to handle intensiometric thermometer that will be a powerful tool for monitoring changes in temperature at a subcellular level and in real-time. In future, the DOFL approach coupled with the infra-red laser microscopic system will further identify a variety of organelle targetable thermometers. Temperature imaging at multiple types of organelles simultaneously will provide a more precise view of heat production, dissipation and transfer at a subcellular level, as well as shed light on the crosstalk between each organelle and individual cells.

## Methods

### DOFL screening in vitro

The DMSO stock solutions (1 mM) of 1500 dyes were diluted to 2.5 µM in HEPES buffer solution (20 mM, pH = 7.4) containing 0.5% DMSO and were loaded on 96 well plates. The fluorescence intensity was measured using a Spectra Max Gemini XSF plate reader. Before measurement, the plate loaded with samples was kept at 32°C or 37°C for 1 hr until the temperature reached the stable state. The heating and cooling process from 32°C to 37°C was repeated twice. Detailed methods for screening for temperature sensitive dyes is described in the supplementary information.

### Synthesis and characterization of ER thermo yellow

Aminophenyl bodipy (10 mg, 32 µmole) and 3-threefluoromethoxy-6-hydroxy-benzaldehyde (9.1 mg, 48 µmole) were dissolved with acetonitrile (5 mL) in a 20 mL vial, and 3 eq. of pyrrolidine and acetic acid were then added to the reaction mixture. The reaction vial was heated to 60°C using a heating block reactor for 3 hrs. After completion of the reaction, NaHCO_3_ saturated solution (50 µL) and chloroacetylchloride 20 µL were added to the reaction mixture. The crude compound was purified by column chromatography and the final product was 15 mg (81%). ^1^H-NMR (CD_3_OD + CDCl_3_) δ 7.67(s, 1H), 7.66(s, 3H), 7.62(s, 1H), 7.42(bs, 1H), 7.30(d, J = 8.3 Hz, 2H), 6.98(d, J = 8.6 Hz, 1H), 6.76(d, J = 8.8, 1H), 6.72(s, 1H), 6.34(m, 2H), 4.13(s, 2H), 1.56(s, 3H), ^13^C-NMR (CD_3_OD + CDCl_3_) δ 158.34, 154.45, 145.65, 142.04, 140.94, 138.07, 134.93, 133.90, 130.40, 130.39, 129.75, 126.22, 124.07, 123.39, 121.49, 120.14, 119.56, 119.44, 119.05, 116.53, 116.04, 53.30, 42.78, 29.53, 15.25; ESI-MS m/z (M + H) calc'd: 576.12, found 576.0, and 556.0(M - F).

### Staining procedures and microscopic experiments

For all microscopic experiments, to evaluate temperature sensitivity, 0.5 µL of ER thermo yellow DMSO stock solution (1 mM) was added into 2 mL of pre-warmed culture medium and then incubated at 37°C, 5% CO_2_ for 30 minutes. After incubation, washing with fresh pre-warmed medium was carried out once. Regarding fixed HeLa cells, after staining with ER thermo yellow, they were treated with 4% formaldehyde solution for 30 min at room temperature and washed three times with 20 mM HEPES buffer solution (pH = 5.0, 6.0, 7.0 and 8.0). For co-staining experiments, the final concentration of ER tracker Green, DAPI, and Fluo 4 were 500 nM, 1 µM, and 1 µM, respectively. All microscopic experiments were carried out at a temperature of 37°C, maintained by a circulation chamber. Microscopic images using ER tracker Green (or Fluo 4), DAPI, and ER thermo yellow (or ER tracker red) were obtained with FITC (488 nm laser), DAPI, Texas red (543 nm laser) filter sets. The optical setup was built around the inverted microscope (IX81, Olympus), and the facility to create local and transient temperature gradients was largely the same as previously reported^7^. A near infrared laser beam (1064 nm, KPS-KILAS-COOL-1064-02-P, Keopsys) was focused using an objective into an aluminum aggregate fixed at the tip of a glass micro needle. The glass micro needle was positioned using a three axis motorized micromanipulator (EMM-3NV, Narishige). For imaging of the live cells, a 561 nm laser (Sapphire 561, Coherent) was guided into a spinning disc confocal unit (CSU-10, yokogawa) attached to the left side port of the microscope. The photobleaching correction was not executed for the data analysis in Figure 2 and 3 because it was not significant. The concurrent observation of heat production and cytosolic Ca^2+^ concentration was carried out with a confocal laser microscope (FV1000 IX81, Olympus). In order to avoid crosstalk resulting from the strong fluorescence of Fluo 4 and ER thermo yellow, the emission channel for ER thermo yellow was restricted to the tail end, i.e., between 590 and 690 nm. Photobleaching could not be ignored in this setup and therefore was corrected by fitting to a single exponential curve (Fig. 4 and Supplementary Fig. S10). A stock solution of 100 µl (20 µM ionomycin, DMSO 0.05% content) was added to 2.0 ml DMEM without phenol red, and was added at 100 sec. The fluorescence images were recorded at 1.8 sec sequentially. For all resulting microscopic images, photobleaching was corrected by fitting to an exponential curve.

### Cell culture and differentiation

HeLa cells were cultured in Dulbecco's modified eagle's medium (DMEM) (Invitrogen, CA, USA) supplemented with fetal bovine serum (10%) and penicillin–streptomycin. Cells were grown in a 3.5 cm glass-based dish at 37°C in the presence of 5% CO_2_. The C2C12 myoblast cell line was obtained from American Type Culture Collection (Rockville, MD). Undifferentiated myoblasts were grown in Dulbecco's modified eagle medium (DMEM) supplemented with penicillin (100 U/mL), streptomycin (100 µg/mL), and 10% fetal bovine serum (FBS). When myoblast culture reached 100% confluence, myoblasts were stimulated to differentiate by replacing the medium with DMEM supplemented with penicillin (100 U/mL), streptomycin (100 µg/mL), and 2% heat-inactivated horse serum. Differentiation was allowed to continue for 5 days to obtain differentiated myotubes, with media replacement every 2 days. NIH3T3 and Chang cells were cultured according to methods similar as those used with HeLa cells. Brown adipose tissue was collected from mice dissection. The animal experiment procedures were performed in accordance with a protocol approved by the Institutional Animal Care and Use Committee (IACUC).

### General procedures

All reactions were performed in oven-dried glassware under a positive pressure of nitrogen. Unless otherwise noted, starting materials and solvents were purchased from Aldrich and Acros organics and used without further purification. Analytical TLC was carried out on a Merck 60 F254 silica gel plate (0.25 mm layer thickness) and visualization was carried out with UV light. Column chromatography was performed on a Merck 60 silica gel (230–400 mesh). NMR spectra were recorded on a Bruker Avance 300 NMR spectrometer. Chemical shifts are reported as δ in units of parts per million (ppm) and coupling constants are reported as a J value in Hertz (Hz). The mass of all the compounds was determined by LC-MS of Agilent Technologies with an electrospray ionization source. Spectroscopic measurements were performed on a fluorometer and UV/VIS instrument, Synergy 4 from Bio-Tek and Gemini XS fluorescence plate reader, repsectively. The slit width was 1 nm for both excitation and emission. Relative quantum efficiencies were obtained by comparing the areas under the corrected emission spectrum. The following equation was used to calculate quantum yield: 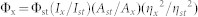


Where Φ_st_ is the reported quantum yield of the standard, I is the integrated emission spectrum, A is the absorbance at the excitation wavelength, and *η_x_* is the refractive index of the solvents used. The subscript x denotes unknown and st denotes standard. Rhodamine B was used as standard.

## Author Contributions

S.A., M.S. and Y.-T.C. designed research. M.S. constructed the microscopic system with infrared laser and S.A. performed the microscopic experiments. S.-C.L. and D.Z. synthesized dyes and tested cell viability. S.A., M.S. and Y.-T.C. wrote this paper. Y.-T.C. organized this study.

## Supplementary Material

Supplementary Information Supplementary information

Supplementary Information Supplementary movie
